# Symptom Burden, Survival and Palliative Care in Advanced Soft Tissue Sarcoma

**DOI:** 10.1155/2011/325189

**Published:** 2011-12-11

**Authors:** Nicholas J. Gough, Clare Smith, Joy R. Ross, Julia Riley, Ian Judson

**Affiliations:** ^1^Palliative Care Department, Royal Marsden Hospital, Fulham Road, London SW3 6JJ, UK; ^2^Division of Medicine, Institute of Cancer Research, 123 Old Brompton Road, London SW7 3RP, UK; ^3^National Heart and Lung Institute, Imperial College, London SW7 2AZ, UK; ^4^Sarcoma Unit, Royal Marsden Hospital, Fulham Road, London SW3 6JJ, UK

## Abstract

*Introduction*. The symptom burden and role of palliative care (PC) in patients with advanced soft tissue sarcoma (STS) are not well defined. *Methods*. This study retrospectively reviewed both symptoms and PC involvement in patients known to an STS referral centre who died in one calendar year. *Results*. 81 patients met inclusion criteria of which 27% had locally advanced disease and 73% metastases at initial referral. The median number of symptoms was slowly progressive ranging from 2 (range 0–5) before first-line chemotherapy (*n* = 50) to 3 (range 1–6) at the time of best supportive care (BSC) decision (*n* = 48). Pain and dyspnoea were the commonest symptoms. Median overall survival from BSC decision was 3.4 weeks. 88% had PC involvement (either hospital, community, or both) with median time from first PC referral to death of 16 (range 0–110) weeks. *Conclusions*. Patients with metastatic STS have a significant symptom burden which justifies early PC referral. Pain, including neuropathic pain, is a significant problem. Dyspnoea is common, progressive and appears to be undertreated. Time from BSC decision to death is short, and prospective studies are required to determine whether this is due to overtreatment or very rapid terminal disease progression.

## 1. Introduction

Soft tissue sarcomas are malignant tumours of connective tissue comprising over 50 different histological subtypes which vary in their clinical behaviour and response to treatment [1, 2]. Surgery, often supplemented by adjuvant radiotherapy, offers the only reliable chance of cure for localised disease [2, 3]; however, over 50% of soft tissue sarcoma (STS) patients will develop metastases [4, 5]. Whilst metastasectomy is increasingly possible [6], palliative treatment generally consists of radiotherapy for locally advanced “inoperable” recurrence and systemic chemotherapy for widespread metastatic disease [1–3]. The aim of such palliative treatments is to establish disease control thus improving survival and symptomatology [2]. 

Median overall survival (OS) from commencing first- and second-line palliative chemotherapy is reported as 12 months [7, 8] and 8 months [9], respectively. Systemic chemotherapy has the potential for significant toxicity [10], and whilst this is routinely recorded as part of clinical trials [11], there is a paucity of generalised STS symptom prevalence data. A recent study of the STS population as a whole in one United Kingdom (UK) sarcoma unit found a pain prevalence of 53% at the time of assessment of which 63% was described as inadequately controlled [12]. 

Disease- or treatment-related symptoms are frequently managed by oncologists; however, more complex symptom control can be challenging and require specialist input. Given the potential for symptoms and limited prognosis, there would seem a clear role for palliative care (PC) team involvement in the advanced STS population. 

Palliative care is defined by the World Health Organisation (WHO) as “an approach that improves the quality of life of patients and their families facing the problems associated with life threatening illness, through the prevention and relief of suffering by means of early identification and impeccable assessment and treatment of pain and other problems, physical, psychosocial and spiritual” [13]. PC teams in the UK provide a spectrum of services including (i) hospital advice/support teams, (ii) hospices providing admissions for symptom control, respite, or end of life care, and (iii) community PC teams who assess and treat patients in their own homes. 

Within UK health care policy, the 2006 National Institute for health and Clinical Excellence (NICE) guidance *Improving outcomes for people with sarcoma* found no specific evidence supporting the role of PC teams in patients with sarcoma [14]. However, it suggested much of its guidance in the 2004 document *improving supportive and palliative care for adults with cancer* [15] was applicable. Specifically, this recommends the effectiveness of specialist PC team involvement for the control of pain and cancer symptoms. It did not, however, suggest when or if PC referral for symptom control or holistic support might be appropriate.

Early PC team involvement has been shown to improve quality of life, mood, and survival in patients with newly diagnosed metastatic non-small-cell lung cancer, a condition with a similar prognosis to metastatic STS [16]. However, in many instances PC is delivered too late to be effective [17, 18]. 

There are anecdotal reports by both STS clinicians and patients that, despite advanced disease, STS patients maintain a good quality of life with moderate symptoms until a rapid decline to the final weeks [19]. Although there are no data to support this, the deterioration has been suggested to differ from the more “predictable” gradual deterioration experienced by those with other cancers such as non-small cell-lung cancer [19]. If true, one might expect that PC team referrals might occur too late to be of benefit to the STS population. It is important to evaluate symptom burden and PC input in locally advanced and metastatic STS to provide recommendations for optimal timing of PC involvement.

This paper presents the results of a retrospective review of physical symptoms and PC team involvement in patients with locally advanced “inoperable”/metastatic STS treated at one tertiary referral centre in the UK.

The aims were to better define the number and severity of physical symptoms at the time of each new treatment decision, for example, before first-line chemotherapy, before second-line chemotherapy, and so forth for locally advanced/metastatic STS. We also wanted to establish the most common symptoms in each group, and the proportion of patients referred to a PC team prior to death along with their OS from the time of diagnosis with metastatic disease.

## 2. Materials and Methods

The records of all patients with a histological diagnosis of locally advanced/metastatic STS over the age of 18, known to the unit and considered for palliative chemotherapy who died during the 2009 calendar year, were analysed. Patients were excluded from the analysis if management did not include palliative chemotherapy assessment (those treated with surgery or palliative radiotherapy alone), if the STS unit only provided a treatment opinion and if the death was considered unrelated to the STS diagnosis. Patients with Gastro-Intestinal Stromal Tumours (GIST) were also excluded as in this well-defined subgroup treatment with molecularly targeted agents such as imatinib can provide a long-term survival benefit.

Data were collected from the hospital electronic patient records and a hand search of paper notes. Missing data from hospital records were obtained from the patient's primary care team.

Each patient's records were analysed from first referral with advanced disease to death. Data collected included demographic information, tumour-specific data, treatment decisions, documented symptoms, and information relating to PC involvement.

More specifically, documented physical symptoms were recorded from the notes prior to each new treatment decision, for example, before first line palliative chemotherapy and were recorded in four categories; “present controlled”, “present uncontrolled”, “documented absent”, or “not documented”. 

The term symptom burden can be defined as symptoms experienced by the patient as a result of the disease itself or associated treatments [20]. 

In this study, we assessed clinician documented physical symptoms prior to the start of a new treatment decision. The impact of systemic therapy on symptom burden was not directly studied. Overall survival (OS) was measured from the start of each new treatment decision until death. Permission from the clinical audit committee was obtained prior to data collection.

## 3. Results

**Figure 1:**
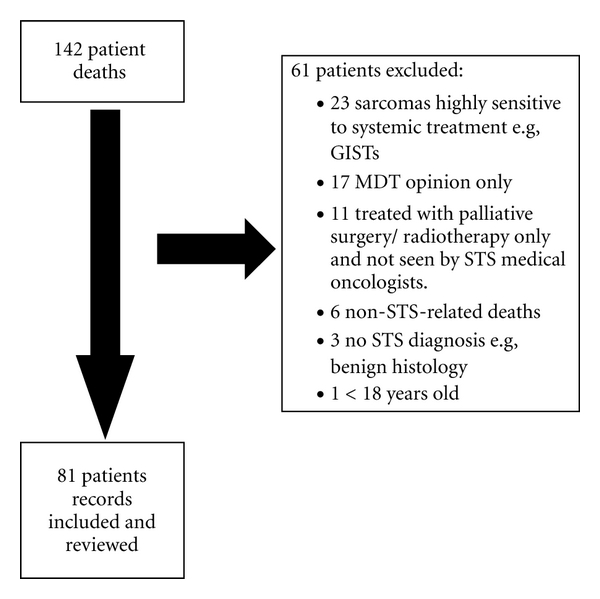
Profile of patients reviewed.

One hundred and forty-two STS patients with locally advanced/metastatic disease known to the STS unit died during the review period 1st January 2009–31st December 2009. Sixty-One patients did not meet the inclusion criteria for review (see Figure 1) resulting in a total of eighty-one patient records analysed.

### 3.1. Demographics and Tumour-Specific Information

The demographic- and tumour-specific details of these patients are described in Table 1. Thirty-five patients (43%) were male with a median age at death of 55 years, range from 18 to 84. Seventy-six patients (94%) presented with “new” advanced “inoperable”/metastatic disease and 5 (6%) had already received treatment for advanced disease in other oncology centres prior to review by the STS medical oncology unit. Fifty-nine patients (73%) had metastatic disease at referral, with 17 (29%) having multiorgan disease.

One hundred and fifty-six treatment decisions were made for the 81 patients and the notes reviewed prior to each of these decisions. Fifty patients received first-line chemotherapy, 28 second line, 15 third line and 7 fourth line. Eight patients were referred for phase 1 drug trials, and 48 patients had a best supportive care (BSC), that is, no further active treatment decision made by the STS unit. 

In addition, seven patients (9%) underwent metastasectomies after favourable responses to chemotherapy. 18 (22%) received palliative radiotherapy at some point after referral with the documented aims being reduction in primary tumour size (7 patients), analgesia (5 patients), treatment of brain metastases (5 patients) and treatment of spinal cord compression (1 patient).

### 3.2. Symptom Burden

**Figure 2:**
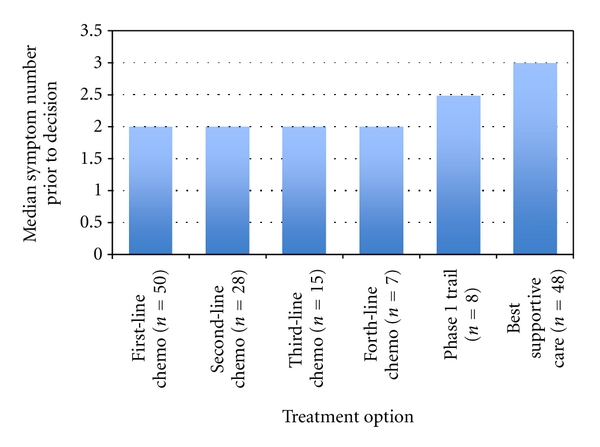
Median number of symptoms prior to each treatment decision.

The median number of symptoms documented prior to each new treatment decision ranged from 2 at the time of first-line chemotherapy to 3 at BSC (Figure 2).  Table 2 shows all documented symptoms at the time of each new treatment decision: pain, dyspnoea, and nausea/vomiting are the three commonest. Other symptoms include fatigue, constipation, and cough. Both figures show that before different lines of chemotherapy, symptom burden was consistent but increased prior to both decision to refer to the Phase 1 trial unit (median 2.5 symptoms) and a best supportive treatment decision (median 3). The two most common documented symptoms were pain and dyspnoea.

**(a) Pain**

Pain was the most common symptom across all treatment decisions/stages of disease. Fifty percent of patients starting first-line chemotherapy experienced pain; however, the proportion of patients with pain rose to 82% (23/28) at second-line chemotherapy and remained similar at BSC decision (79%, 38/48). Twenty percent (10/50) of patients were documented as having uncontrolled pain at first-line chemotherapy compared to 48% (23/48) of patients at BSC decision (Table 3). The gold standard for the effective management of cancer pain is to follow the WHO 3-step analgesic ladder [21].  Table 4 describes the overall use of analgesia in these patients. It shows 86% (70/81) of patients were using a regular “step 1” analgesic, for example, paracetamol a median of 40 weeks before death, whereas 64% (52/81) required a regular “step 3” analgesic—for example, a strong opioid, such as oral morphine a median 14 weeks before death. Interestingly, 28% (23/81) were prescribed a neuropathic agent such as gabapentin, implying that the proportion experiencing neuropathic pain was at least 28%.

**(b) Dyspnoea**

Dyspnoea was the second most common symptom across all treatment decisions/stages of disease except at first-line chemotherapy. Twenty percent of patients starting first-line chemotherapy experienced dyspnoea; however, the proportion of patients rose to 39% (11/28) at second-line chemotherapy, and rose further (44%, 21/48) at BSC decision (see Table 2). This correlates with lung being the most common site of STS metastasis. Six percent (3/50) of patients were documented as having uncontrolled dyspnoea at first-line chemotherapy compared to 31% (15/48) of patients at BSC decision. Overall, medications specifically documented for palliation of dyspnoea (opioids or benzodiazepines) were prescribed in only 15% (12/81) of patients, suggesting that this symptom is undertreated.

### 3.3. Overall Survival

Median OS from first referral irrespective of treatment (*n* = 81) was 38.7 weeks (Table 5) indicating the relatively poor prognosis of this STS cohort. The median OS times from start of first- and second-line chemotherapy mirror established data [7–9]. Of the 59% with a documented BSC decision, OS from decision was 3.4 weeks (range 1–62).

### 3.4. Palliative Care Team Involvement

71 patients (88%) had a PC team referral made either to the hospital team alone (7/71), a community team alone (26/71), or both (38/71). The median time before death from first PC team referral was 15.8 weeks (range 0.1–110.3).

## 4. Discussion

Patients with locally advanced/metastatic STS generally undergo chemotherapy to palliate not cure. This paper shows that these patients experience a significant symptom burden that can be difficult to control. The authors, hope these symptom prevalence data are generalisable and therefore, of value to oncologists treating STS. 

The median number of documented symptoms ranged from 2 at first-line chemotherapy to 3 at BSC decision suggesting sustained and slowly progressive symptoms. The prevalence of documented pain before different palliative treatment decisions was consistently above 50%. This correlates with a systematic review by van den Beuken-van Everdingen et al. suggesting pain prevalence to be 64% in those with advanced/metastatic/terminal cancer of any type [22]. Furthermore, a recent study investigating pain prevalence in the STS population as a whole found a prevalence of 53% [12] of which 36% were found to have neuropathic pain. Whilst documented interpretation of pain type was not recorded, 28% of patients in this paper were prescribed neuropathic analgesic agents correlating with this recent published data. 

Dyspnoea can be multifactorial in aetiology; however, the high prevalence of documented breathlessness correlates with lung as the commonest site of metastases in STS. At referral, 38 of the 59 patients (64%) with metastatic disease had lung metastases; this is comparable to findings from other studies [7, 8, 23]. The increasing prevalence of dyspnoea through lines of chemotherapy likely reflects disease progression in the lungs. The small number of patients on a medication specifically to palliate dyspnoea (12 patients) may represent clinicians' lack of confidence in treating this symptom or a lack of documentation clarifying why these drugs (e.g., opiates or benzodiazepines) were prescribed. 

One striking statistic is that those who had a BSC decision (48/81) had a median OS of only 3.4 weeks. This may suggest “active” treatment is being continued late into the disease trajectory, against recommendations arising from a national UK report reviewing deaths within 30 days of receiving systemic anticancer therapy [24]. Conversely, it may also add support to the anecdotal observation that STS patients remain relatively well with good quality of life until late into their illness before a rapid deterioration towards the terminal phase [19].

Importantly, the median OS for all patients was significantly less than one year. The UK Department of Health End of Life Care Strategy [25] advocates the importance of individualised care plans and PC involvement in the last year of life. Given this policy, all the patients in this paper should have had some PC involvement. Encouragingly, 88% of patients were referred to a PC team, with a high proportion of these (64/71, 90%) known to community PC teams. Although not reviewed, this may have enabled advanced care planning such as a patients preferred place of care and death to be established and facilitated. The median time from first PC referral to death of 15.8 weeks may suggest patients at this centre are being referred early enough to potentially benefit from the PC service. The data also suggest that the majority of patients experience symptoms earlier, often from the initial diagnosis of locally advanced/metastatic disease which, therefore, adds further weight to considering PC sooner. 

There are no guidelines at this centre regarding the appropriateness or timing of referral to a PC team. Decisions to refer are made on an individual basis by the oncology team or general practitioner after patient consultation. A nationwide survey of American doctors suggested 13 weeks before death as the most appropriate time to refer to a hospice care program [26]. However, studies in America and other countries have shown physicians refer cancer patients to PC teams/hospice care programs much nearer death—with median survival from initial referral ranging from 3 to 8 weeks [27–32]. Barriers to early PC referrals include (i) limitations in PC access between and within countries, (ii) reluctance of patients/families to be referred because of misunderstandings of what PC may offer/its perceived association with imminent death, and (iii) resistance by clinicians to refer patients still having “active” treatment/their reluctance to discuss end of life issues [33–35]. Interestingly, our data show that 48% (23/48) of patients in this review had documented uncontrolled pain and 31% (15/48) had uncontrolled dyspnoea at the time of a BSC decision. Most had already been referred to a PC team by this point. This may indicate inadequate access to PC services despite referral, or that a rapid escalation in “difficult to control” pain/dyspnoea is a predictive factor for the terminal phase in STS patients. 

The authors recognise these data are from a single UK cancer center. Results will be indicative of the STS units individual practice as well as UK health policy which limits generalisation nationally or globally. Other countries may have different protocols and treatment thresholds which might influence results. That said, OS data from first- and second-line chemotherapy were similar to those of larger cohorts [7–9]. 

The availability of PC services varies within the UK [36] and greatly between/within other European and western countries [37–41] reflecting the heterogeneity of health care systems, patient's needs and cultures. The UK has a relatively active PC network and its government has recently invested in PC resources and end of life care [25, 42]. 

The studies retrospective design is also a potential source of major bias: the symptom data are based on clinician documentation where factors such as inadequate assessment, time pressure, and selective documentation of positive findings may all contribute to inaccuracy. Recording symptoms prospectively using validated patient reported outcome measures may lead to more accurate assessment/outcomes [43]. Patients whose death was not thought attributable to STS were excluded: this is difficult to establish; therefore, all deaths should have been analysed.

## 5. Conclusions

Locally advanced “inoperable”/metastatic STS patients have a significant symptom burden which is slowly progressive and commonly includes pain and dyspnoea. The level and timing of PC team referrals in this UK single centre evaluation was encouraging. However, pain was documented as uncontrolled in 48% of patients at the time of first-line chemotherapy and patients had at least two symptoms at the time of all treatment decisions. There was also a suggestion that dyspnoea was undertreated. The short time from documented BSC decision to death is a concern: this could suggest that patients continue active treatment too long, or that this is due to extremely rapid disease progression in the terminal phase. 

Given the prevalence of symptoms, potential for treatment toxicity, and poor OS, prospective quality of life data could aid decision making in the STS population. Given the potential for PC to improve quality of life and survival in patients with advanced cancer, these data support the need for early PC referral in patients with metastatic STS. The lack of prospective studies into this important area indicates the need for further research.

## Figures and Tables

**Table 1:** Demographics and tumour-specific details.

Demographic- and tumour-specific factors:	Number	**%**
Number:	81	
Male	35	43.2
Female	46	56.8
Median age at death (Range)	55 (18–84)	
Histology:		
Leiomyosarcoma	23	28.4
Liposarcoma	12	14.8
Angiosarcoma	7	8.6
Synovial sarcoma	6	7.4
Sarcoma—(Not other specified)	6	7.4
Other	27	33.4
Disease status at referral:		
Locally advanced/“inoperable”	22	27.2
Metastatic	59	72.8
Metastasis at referral:		
Single organ	42	71.2
Multiple organ	17	28.8
Site of metastases at referral:		
Lung	38	64.4
Liver	12	20.3
Soft tissue	15	25.4
Bone	9	15.3
Other	9	15.3

**Table 2:** All documented symptoms prior to different palliative treatment decisions. (Due to the small numbers, only documented symptoms at the time of first- and second-line chemotherapy and best supportive care decision are displayed).

Symptom	First-line palliative chemotherapy (*n* = 50)	Second-line palliative chemotherapy (*n* = 28)	Best supportive care (*n* = 48)
	Symptom prevalence	Symptom prevalence	Symptom prevalence
Pain	25 (50%)	23 (82%)	38 (79%)
Breathlessness	10 (20%)	11 (40%)	21 (44%)
Nausea and vomiting	11 (22%)	5 (18%)	17 (35%)
Fatigue	9 (18%)	5 (18%)	16 (33%)
Constipation	6 (12%)	2 (7%)	8 (17%)
Cough	3 (6%)	3 (11%)	9 (19%)
Feeling bloated	9 (18%)	2 (7%)	3 (6%)
Weight loss	6 (12%)	0 (0%)	1 (2%)
Low appetite	4 (8%)	0 (0%)	9 (19%)
Diarrhoea	1 (2%)	0 (0%)	4 (8%)
Dry mouth	1 (2%)	1 (4%)	3 (6%)
Trouble sleeping	2 (4%)	0 (0%)	4 (8%)
Numbness/tingling in hands/feet	0 (0%)	1 (4%)	3 (6%)
Problems with urination	2 (4%)	0 (0%)	2 (4%)
Sweats	0 (0%)	0 (0%)	1 (2%)

**Table 3:** The documentation of symptoms. (Due to the small numbers, only the three commonest documented symptoms at the time of first- and second-line chemotherapy and best supportive care decision are shown here).

Symptom	First-line palliative chemotherapy (*n* = 50)	Second-line palliative chemotherapy (*n* = 28)	Best supportive care (*n* = 48)
Pain documented as:			
Present controlled	15 (30%)	19 (68%)	15 (31%)
Present uncontrolled	10 (20%)	4 (14%)	23 (48%)
Absent documented	15 (30%)	2 (7%)	7 (15%)
Not recorded	10 (20%)	3 (11%)	3 (6%)
Breathlessness documented as:			
Present controlled	7 (14%)	10 (36%)	6 (13%)
Present uncontrolled	3 (6%)	1 (4%)	15 (31%)
Absent documented	20 (40%)	8 (28%)	20 (43%)
Not recorded	20 (40%)	9 (32%)	7 (15%)
Nausea and Vomiting documented as:			
Present controlled	8 (16%)	4 (14%)	15 (31%)
Present uncontrolled	3 (6%)	1 (4%)	2 (4%)
Absent documented	21 (42%)	15 (54%)	21 (44%)
Not recorded	18 (36%)	8 (28%)	10 (21%)

**Table 4:** Symptom control drug use.

	WHO Class 1 Analgesic, for example, Paracetamol	WHO Class 2 Analgesic, for example, Codeine	WHO Class 3 Analgesic, for example, Morphine sulphate	Agent specified for neuropathic pain, for example, Gabapentin	Agent specified for dyspnoea, for example, Lorazepam
Patients using	70	52	52	23	12
%	86	64	64	28	15
Median time started before death in weeks (Range)	39.7 (1–202)	32.3 (2–206)	13.9 (1–106)	11.1 (1–83)	3.4 (1–56)

**Table 5:** Overall survival.

	Overall	First-line chemo	Second-line chemo	Third-line chemo	Fourth-line chemo	Phase 1 drug trial	Best supportive care
Number of patients	81	50	28	15	7	8	48
Overall survival in weeks (Range)	38.7 (1–212)	48.6 (3–200)	43.0 (1–151)	15.8 (5–100)	13.6 (9–27)	14.9 (1–46)	3.4 (1–62)
